# Comparison of survival of patients receiving laparoscopic and open radical resection for stage II colon cancer

**DOI:** 10.2478/v10019-011-0029-0

**Published:** 2011-09-22

**Authors:** Cui-Zhen Fan, Yu-Ping Chu, Ping Wei, Hong Dai, Wenming Chen

**Affiliations:** 1 Department of Oncology, Beijing Chaoyang Hospital, Capital University of Medical Science, Beijing, China; 2 Department of Pathology, Beijing Chao yang Hospital, Capital University of Medical Science, Beijing, China; 3 Department of Hematologic Neoplasms and Oncology, Beijing Chaoyang Hospital, Capital University of Medical Science, Beijing, China

**Keywords:** stage II colon cancer, laparoscopy, chemotherapy, prognosis

## Abstract

**Background:**

The aim of the study was to compare the survival of patients receiving laparoscopic *vs.* open radical resection for stage II colon cancer.

**Patients and methods:**

Two hundred and twenty patients with stage II colon cancer were enrolled from Beijing Chaoyang Hospital of Capital Medical University from January 2000 to December 2009, including 61 patients in the laparoscopic radical resection group and 159 patients in the open radical resection group. The survival data in both groups were compared using the log rank test based on Kaplan-Meier survival curves.

**Results:**

There was no statistically significant difference in the 3-year survival (88.5% *vs.* 80.5%; X^2^=1.98, *P*=0.159) and the 5-year survival (81.9% *vs.* 69.2%; X^2^=1.98, *P*=0.159) between both groups. However, statistically significant difference was found in median overall survival (mOS), which was 102.6 (95% CI: 76.8–122.7) months in the laparoscopic group and 90.0 (95% CI: 70.4–109.6) months in the open radical resection group (X^2^=4.183, *P*=0.041). mOS was 96 (95% CI: 68.6–111.4) months and 92.6 (95% CI: 56.8–107.2) months in those with and without postoperative chemotherapy, respectively (X^2^=6.389, *P*=0.011). For patients older than 75 years the mOS was 90.0 (95% CI: 25.3–105.0) months and 83.4 (95% CI: 13.1–96.9) months in the laparoscopic and open group, respectively. The difference between the both groups was statistically significant (X^2^=6.191, P=0.013).

**Conclusions:**

The mOS of patients receiving laparoscopic radical resection was better than open radical resection for stage II colon cancer, especially for patients over 75 years old.

## Introduction

The incidence of colorectal cancer is 3.6–59.1 per 100,000 people worldwide.^1^ It is one of the most common malignancies in the world. Its incidence is still increasing as people’s lifestyle changes; especially in developing countries.^2^,^3^ Surgical resection is still the only approach for curing colorectal cancer. The gold criterion of successful removal is that the cancer margins and lymph nodes in relative regions are completely resected. Currently there are many reports available on laparoscopic radical resection for colorectal cancer. Laparoscopic radical resection achieves rapid recovery and few postoperative complications with recognized short-term outcomes better than open radical resection.^4^–^7^ Latest follow-up data of laparoscopic radical resection also confirm the long-term outcomes of laparoscopic radical resection for colorectal cancer; the 1-year, 3-year, and 5-year survival following laparoscopic radical resection is similar to that following open radical resection.^5^–^7^ However, the survival might depended on post-treatment surveillance of patients.^8^ There is also report on better efficacy of laparoscopic radical resection than open radical resection as laparoscopic radical resection reduces cancer recurrence, cancer-related mortality and other risks.^9^ In the present study, the survival of patients receiving laparoscopic and open radical resection for stage II colon cancer in 220 patients with stage II colon cancer enrolled from Beijing Chaoyang Hospital of Capital Medical University between January 2000 and September 2009 were retrospectively compared.

## Patients and methods

Two hundred and forty-nine patients with stage II colon cancer were treated in Beijing Chaoyang Hospital of Capital Medical University from January 2000 to December 2009. Twenty-nine patients were lost during the follow up. Two hundred and twenty of them were included into the present study according to the inclusion criteria. There were 61 in the laparoscopic radical resection group and 159 in the open radical resection group. The inclusion criteria were: (a) complete medical records with definitive pathology diagnosis of colon cancer treated with radical resection; (b) stage II in the TNM staging system and neoadjuvant chemotherapy not practiced. Exclusion criteria were: (a) synchronous or metachronous colorectal carcinoma, or familial adenomatous polyposis; (b) multiple primary malignant tumours; (c) surgery complication related death; and (d) laparoscopic radical resection replaced by open radical resection. Informed consent was obtained from all these patients. Sixty-four patients underwent postoperative 5-fluorouracil based chemotherapy, while 156 patients were only underwent radical resection.

Preparations prior to laparoscopic and open radical resection were similar. Tracheal catheterization and general anaesthesia were administered. Surgical procedure was performed according to instructions for tumour-free surgery.

Among 64 patients treated with chemotherapy, fifty-tree patients underwent 5-fluorouracil based chemotherapy, complemented by calcium folinate, cis-platinum and oxaliplatin for 4–6 courses, and 11 patients were treated by xeloda alone or combination of xeloda and oxaliplatin for 6–8 courses.

Evaluation of recurrence of colon cancer comprised physical examination, chest X-ray, abdominal CT, and colonoscopy (once a year). The patients were followed up through telephone, outpatient visits and inpatient records. The follow up started from the day of surgery and ended on December 31, 2010. The end-point-data would be 3, 5-year survival and median overall survival (mOS).

Statistical analyses were done using SPSS 15.0. X^2^ test was performed for general data including age and gender. The Kaplan-Meier survival curves and the log rank test were used to analyse the survival data with the selection of operation and treatment. For all analyses, the level of significance was set at *P*<0.05.

## Results

There was no statistically significant differences in the gender, age, cancer site, histological classification, differentiation, vascular thrombus, nerve invasion, lymph nodes revealed by postoperative pathology, or postoperative chemotherapy between the laparoscopic and open radical resection groups (*P*>0.05) (Table 1).

**FIGURE 1:**
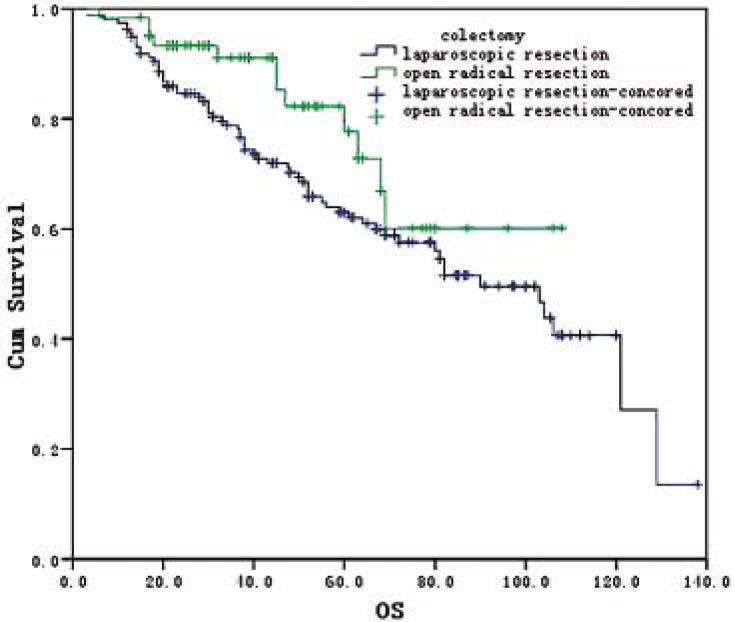
Overall survival curves for 220 patients undergoing laparoscopic and open radical resection.

**FIGURE 2:**
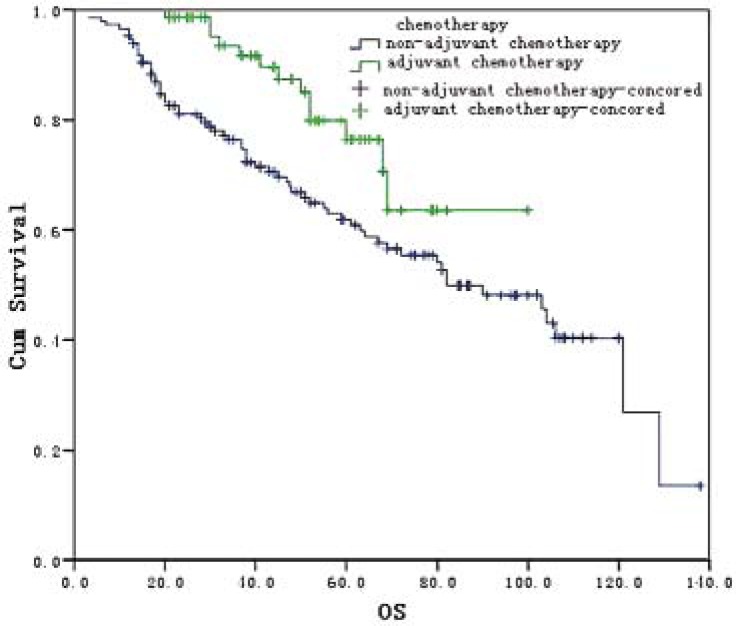
Overall survival curves for 220 patients with or without adjuvant chemotherapy.

Twenty-nine patients were lost to the follow up with a loss rate of 11.6%. The follow up period ranged from 3 to 128 months with an average of 52.5 months. There was no statistically significant difference in the 3-year survival (88.5% *vs.* 80.5%; X^2^=1.98, *P*=0.159) and the 5-year survival (81.9% *vs.* 69.2%; X^2^=1.98, *P*=0.159) between both groups. However, statistically significant difference was found in mOS, which was 102.6 (95% CI: 76.8–122.7) months in the laparoscopic group and 90.0 (95% CI: 70.4–109.6) months in the open radical resection group (X^2^=4.183, *P*=0.041) (Figure 1). mOS was 96 (95% CI: 68.6–111.4) months and 92.6 (95% CI: 56.8–107.2) months in those with or without postoperative chemotherapy, respectively (X^2^=6.389, *P*=0.011) (Figure 2).

**FIGURE 3:**
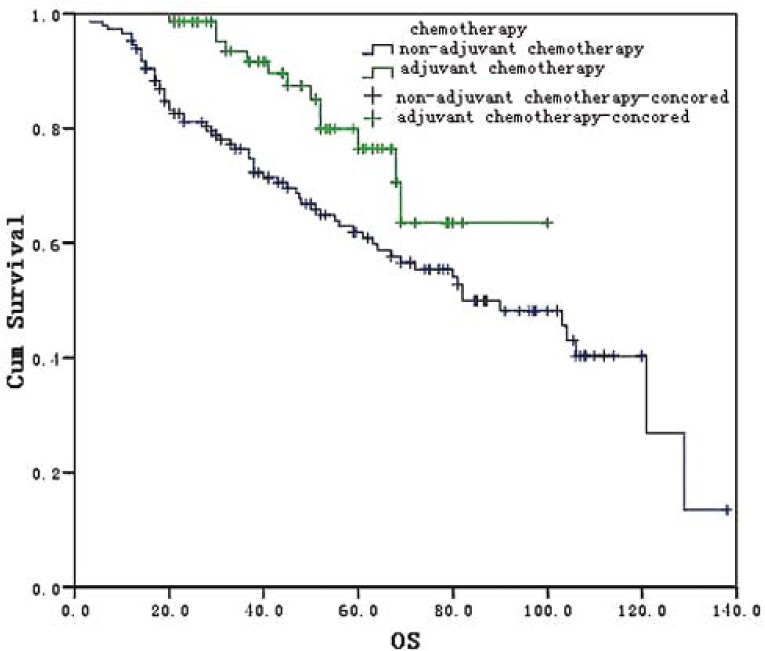

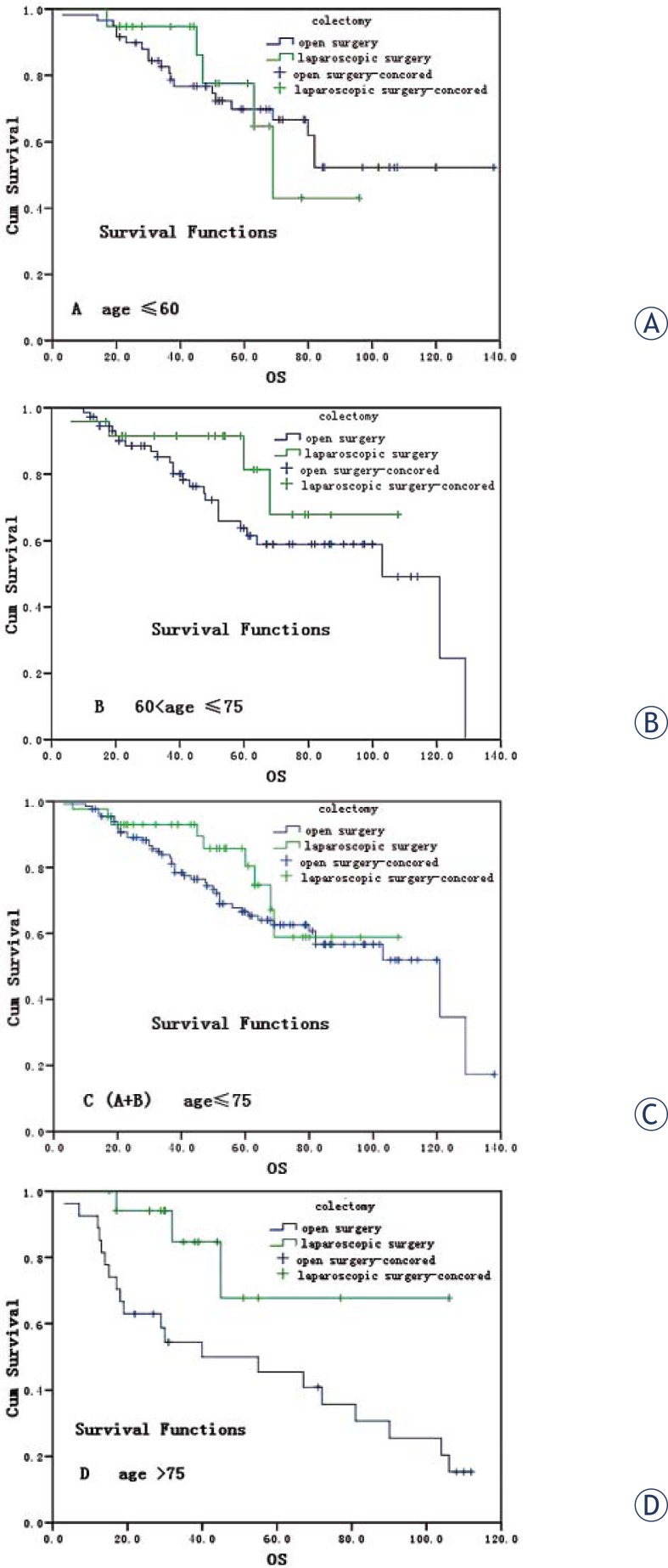
Survival curves for 220 patients undergoing laparoscopic or open radical resection in different age periods: A: less than 60 years old; B, 60–75 years old; C=A+B; D, 75 years old.

For patients below 75 years old, the mOS was 108 (95% CI: 68.9∼173.0) months and 120.8 (95% CI: 69.5∼172.5)90.0 months in the laparoscopic and open radical resection groups, without statistically significant difference (X^2^=1.0136.191, *P*=0.314). For patients older than 75 years the mOS was 90.0 (95% CI 25.3 – 105.0) months and 83.4 (95% CI: 13.1 – 96.9) months in the laparoscopic and open group, respectively. The difference between these two groups was statistically significant (X^2^=6.191, P=0.013) (Figure 3).

## Discussion

Since Jacobs *et al*. reported the initial use of laparoscopic radical resection of sigmoid colon, laparoscopic radical resection has been increasingly used for colorectal cancer.^10^ However, questions are raised regarding whether the long-term outcomes of laparoscopic radical resection are comparative to that of open radical resection and whether it leads to tumour metastasis.

This study showed that the 3-year survival was 88.5% and 80.5% in the laparoscopic and open radical resection groups in 220 patients with stage II colon cancer. Bonjer *et al*.^6^ reported that the 3-year survival was 82.2% and 83.5% respectively for both groups in stage II colon cancer. Kitano *et al.*^11^ found that the 5-year survival was 94.8% for laparoscopic radical resection, comparable to open radical resection. Fleshman *et al*.^7^ reported that the 5-year survival was 74.6% and 76.4% respectively for laparoscopic and open radical resection group in a multi-centre study in 872 patients with colon cancer and concluded that there was no statistically significant difference in the overall survival and the disease-free survival between two groups, suggesting that the long-term efficacy is similar for two procedures. This current study found similar results in stage II colon cancer patients, again confirming that laparoscopic radical resection can achieve favourable outcomes for early-stage colon cancer.

In our study the mOS was 102.6 months and 90.0 months in the laparoscopic and open radical resection groups with a statistically significant difference, further demonstrating laparoscopic radical resection has better survival outcomes than open radical resection. Bilimoria *et al*.^12^ reported that the 5-year survival was apparently better for laparoscopic radical resection in patients with stages I and II colon cancer. Lacy *et al*.^9^ revealed that laparoscopic radical resection reduced cancer recurrence, risks of mortality from cancer, and other risks, and that the tumour-bearing survival was better for laparoscopic radical resection than open radical resection in a long-term follow up of 218 cases of colon cancer. These results may be attributable to minimal invasion of the surgery and rapid rehabilitation of immune function following laparoscopic radical resection.^13^–^15^

The 5-year survival is 75% −80% for stage II colon cancer following radical resection and 20%–25% patients die of recurrence or distant metastasis.^16^ As there are no large-scale clinical trials that conclude that stage II colon cancer patients can benefit from postoperative adjuvant chemotherapy, postoperative chemotherapy is thus controversial for stage II colon cancer.^17^,^18^ The National Surgical Adjuvant Breast and Bowel Project (NSABP) thought that stage II colon cancer patients could benefit from adjuvant chemotherapy as stage III patients.^19^ In this study, the mOS was 96 months for patients with postoperative chemotherapy and 92.6 months for those without chemotherapy with a statistically significant difference, showing that chemotherapy is advantageous whatever surgical technique is adopted.

Although meta-analyses could not substitute large randomised clinical studies^20^, we cannot neglected that a pooled analysis of five randomized trials did not show the radical resection with adjuvant chemotherapy was better than radical resection alone.^21^ Therefore, the National Comprehensive Cancer Network (NCCN) guidelines recommend adjuvant chemotherapy for stage II colorectal cancer patients with risks for poor prognosis (high risks), such as poor histological differentiation, stage T4, invasion to blood vessels or lymph vessels, intestinal obstruction or perforation, tumours too near resection margins, and less than 12 lymph nodes for pathology examination.^22^ Moreover, some proteins are accepted as predictors for adjuvant chemotherapy for high-risk stage II colorectal cancer.^23^ Though this study indicates that chemotherapy was beneficial for patients like in the metastatic diseasse^24^, multi-centre trials with a large sample size and different chemotherapy regimens are required to demonstrate the effect of adjuvant chemotherapy for stage II colon cancer. Additionally, there was no statistically significant difference in the cancer site, histological classification, nerve invasion, lymph nodes, or postoperative chemotherapy between the laparoscopic and open radical resection groups.

About 50% colorectal cancer patients are over 70 years old and colorectal cancer thus becomes a common disease for patients over 70 years old.^25^ According to the Colorectal Cancer Collaborative Group in UK, the risk of surgery for the old increases with age; the mortality was 1.3%–5.2% for patients of 65 years old or above, and 7.1%–8.9% for patients of 85 years or above.^26^ But another study demonstrates that radical resection is safe in old patients with colorectal cancer and high risks of radical resection are mainly correlated with complications and emergency treatment instead of age.^27^ In this study, there were 18 patients over 75 years old in the laparoscopic radical resection group and 27 patients over 75 years old in the open radical resection group. For patients of over 75 years old, it is suggested that the survival of laparoscopic radical resection is superior over open radical resection for stage II colon cancer. The advantage in survival is probably related to less invasive nature of laparoscopic procedure, which can be of greatest benefit in the patients older than 75 years

## Conclusions

The survival of patients receiving laparoscopic radical resection was better than that of open radical resection for stage II colon cancer, especially for patients over 75 years old. Thus laparoscopic radical resection should be selected for these stage II colon cancer patients as well as postoperative adjuvant chemotherapy for better survival.

## Figures and Tables

**TABLE 1:** General information of 220 patients with stage II colon cancer

	**Laparoscopic radical resection**	**Open radical resection**	**X^2^**	**P**
**Total**	61	159		
**Sex**				
Male	28	86	1.183	0.276
Female	33	73		
**Age**				
∼60 years	19	59	4.255	0.119
∼75 years	24	73		
>75 years	18	27		
**Tumor site**				
Ascending colon	20	75	3.910	0.418
Transverse colon	6	14		
Descending colon	10	22		
Sigmoid colon	25	48		
**Pathological classification**				
Highly differentiated adenocarcinoma	1	4	0.608	0.962
Moderately differentiated adenocarcinoma	50	123		
Mucous adenocarcinoma	6	20		
Lowly differentiated adenocarcinoma with signet ring cells	4	12		
**Chemotherapy**				
Yes	23	41	2.485	0.115
No	38	118		
**Vascular thrombus**				
Yes	54	145	0.364	0.546
No	7	14		
**Nerve invasion**				
Yes	54	146	0.1829	0.669
No	6	13		
**Lymph node metastasis**				
≥12	24	90	1.418	0.492
<12	37	69		
